# The Role of KLF2 in the Regulation of Atherosclerosis Development and Potential Use of KLF2-Targeted Therapy

**DOI:** 10.3390/biomedicines10020254

**Published:** 2022-01-24

**Authors:** Siarhei A. Dabravolski, Vasily N. Sukhorukov, Vladislav A. Kalmykov, Andrey V. Grechko, Nikolay K. Shakhpazyan, Alexander N. Orekhov

**Affiliations:** 1 Department of Clinical Diagnostics, Vitebsk State Academy of Veterinary Medicine [UO VGAVM], 7/11 Dovatora Str., 210026 Vitebsk, Belarus; 2 Laboratory of Cellular and Molecular Pathology of Cardiovascular System, AP Avtsyn Research Institute of Human Morphology, 3 Tsyurupy Str., 117418 Moscow, Russia; vasily.sukhorukov@morfolhum.ru (V.N.S.); xxor2011@gmail.com (V.A.K.); 3 Laboratory of Medical Genetics, Institute of Experimental Cardiology, National Medical Research Center of Cardiology, 121552 Moscow, Russia; 4 Laboratory of Angiopathology, Institute of General Pathology and Pathophysiology, Russian Academy of Medical Sciences, 125315 Moscow, Russia; 5 Federal Research and Clinical Center of Intensive Care Medicine and Rehabilitology, 14-3 Solyanka Str., 109240 Moscow, Russia; avg-2007@yandex.ru; 6 Laboratory of Clinical Pathology, Institute of Human Morphology, 3 Tsyurupa Str., 117418 Moscow, Russia; nshakhpazyan@gmail.com; 7 Institute for Atherosclerosis Research, 4-1-207 Osennyaya Str., 121609 Moscow, Russia; a.h.opexob@gmail.com

**Keywords:** atherosclerosis, Kruppel like factor 2, inflammation, atheroprotection, shear stress

## Abstract

Kruppel like factor 2 (KLF2) is a mechanosensitive transcription factor participating in the regulation of vascular endothelial cells metabolism. Activating KLF2 in endothelial cells induces eNOS (endothelial nitric oxide synthase) expression, subsequent NO (nitric oxide) release, and vasodilatory effect. In addition, many KLF2-regulated genes participate in the anti-thrombotic, antioxidant, and anti-inflammatory activities, thereby preventing atherosclerosis development and progression. In this review, we summarise recent evidence suggesting that KLF2 plays a major role in regulating atheroprotective effects in endothelial cells. We also discuss several recently identified repurposed drugs and natural plant-based bioactive compounds with KLF2-mediated atheroprotective activities. Herein, we present a comprehensive overview of the role of KLF2 in atherosclerosis and as a pharmacological target for different drugs and natural compounds and highlight the potential application of these phytochemicals for the treatment of atherosclerosis.

## 1. Introduction

### 1.1. Atherosclerosis and Shear Stress

Atherosclerosis is the primary pathology of CVDs (cardiovascular diseases) and the leading cause of morbidity and mortality around the globe [1]. Mainly, atherosclerosis occurs in arteries that consist of different vascular (such as VSMCs (vascular smooth muscle cells)) and endothelial cells. The development of atherosclerosis begins with endothelial dysfunction and subsequent retention and modification of LDL (low-density lipoproteins), accompanied by adhesion, rolling, and transmigration of leukocytes into the sub-endothelial space. Further, they differentiate into macrophages and uptake different forms of LDL (primarily oxidized forms), leading to the formation of “foam cells” and atherosclerotic plaques [2]. Without proper treatment, such plaque accumulation leads to CVDs and acute events (arterial thrombosis, tissue ischemia, and vessel occlusion, heart attacks, and ischemic strokes) [3,4].

NO (nitric oxide) is a crucial endothelium-derived relaxation agent known as a cardiovascular protecting and anti-atherosclerosis factor. However, under pathological conditions, eNOS (endothelial nitric oxide synthase) produces an abnormally high amount of NO, which could cause endothelial dysfunction and trigger atherosclerosis development. Oxidized LDLs is one of the main factors modulating eNOS/iNOS (inducible NO synthase) machinery and promoting endothelial dysfunction and vascular inflammation [5].

It is well-known that atherosclerotic plaques are more prone to the regions of arteries with DF (disturbed flow), such as arterial bending and branching points. On the other hand, the straight parts of arteries with UF (unidirectional laminar flow) are protected from atherosclerosis [6]. Such distinctions could be explained by the different types of cellular responses, activated in the endothelial cells by hemodynamic forces (from flowing blood) and shear stress (dragging force, acting on the surface of the vascular lumen) [7]. Shear stress modulates the structure and function of endothelial cells, thus, playing a critical role in atherosclerosis and CVDs development [8,9].

Endothelial cells are the major cell type able to sense and transduce mechano-signals from shear stress to regulate different biological functions and signaling pathways. Those effects are facilitated by mechanosensors and mechanotransducer, localized into the membrane of endothelial cells [10]. UF is associated with high laminar shear stress and occurs in straight parts of arteries with steady and uniform blood flow; it provides anti-atherosclerotic and anti-inflammatory effects preserves homeostasis and quiescence of endothelial cells. DF, on the contrary, is known as an irregular and non-uniform flow pattern with a low magnitude of shear stress, associated with many CVDs risk factors (such as hyperglycemia and hyperlipidemia), leading to endothelial dysfunction and promoting the distribution of atherosclerosis plaques [11].

UF and DF provide diverse effects on the endothelial cells, affecting primary biological functions and processes (such as glucose metabolism, mitochondrial functions, cell proliferation and alignment, oxidative stress, and inflammation) mainly through the expression of MSTFs (mechanosensitive transcription factors) [12].

### 1.2. Kruppel like Factor 2

Endothelial KLF2 is the best-characterized member of the KLF family and MSTF in general, which regulates the expression of a wide range of anti-thrombotic, antioxidant, and anti-inflammatory genes in endothelial cells. In addition, UF up-regulates *KLF2*, and DF—downregulates [13].

There are many crucial targets of KLF2, such as *eNOS*, *THBD* (thrombomodulin), number of pro-inflammatory, vasoconstrictive and pro-thrombotic genes (*PAI1* (plasminogen activator inhibitor-1), *SELE* (E-selectin), *ET1* (endothelin 1), *MCP*-*1* (monocyte chemoattractant protein), and *VCAM1* (vascular cell adhesion molecule). In addition, KLF2 recruits transcriptional co-activator p300 to inhibit pro-inflammatory genes [14,15,16,17]. The athero-protective role of KLF2 on aortic vessels is based on the accelerated atherosclerosis when KLF2 and KLF4 deficient mice combined with apolipoprotein E (*ApoE*^−/−^) and LDLR (*LDLR*^−/−^) deficient mice. In particular, *KLF2*^+/−^; *ApoE*^−/−^ mice show an increase in lipid uptake, foam cell formation, and atherosclerotic lesions [18]. *PPAP2B* (phosphatidic acid phosphatase type 2B) and its variant rs17114036 are recently identified flow-sensitive genes regulated by KLF2 and associated with ischemic stroke and coronary artery disease via UF-induced endothelial cell alignment and anti-inflammatory effects [19,20]. Further, UF regulates endothelial cells metabolism in a KLF2-dependent way. In particular, *PFKFB3* (6-phosphofructo-2-kinase/fructose-2,6-biphosphatase-3) is a KLF2 target, which represses glycolysis and mitochondrial respiration, thus, maintaining vascular homeostasis and regulating endothelial cellular metabolism [21].

Further in this review, we focus on the role of KLF2, one of the master regulators of the shear stress-induced gene expression in endothelial cells and the development of atherosclerosis.

## 2. KLF2 as the Main Regulator of the NO Production

Nitric oxide (NO) is one of the most crucial endogenous substances to maintain normal endothelial function vascular homeostasis and regulate vessel tone. In ECs (endothelial cells), NO is known for its highly athero-protective properties through its combined antioxidant, anti-apoptotic, anti-thrombotic, and anti-inflammatory effects, and also for pro-apoptotic and anti-proliferative effects in intimal smooth muscle cells. NO production in ECs is regulated via constitutive expression of *eNOS*, a calcium/calmodulin-dependent enzyme converting L-arginine to L-citrulline in the presence of molecular oxygen [22]. The regulation of *eNOS* expression and function in ECs is complex and regulated by many transcriptional, translational, epigenetic, and post-translational mechanisms. Various stimuli, such as exercise, extracellular ATP, lipoproteins, shear stress, different hormones, and *TGFB1* (transforming growth factor beta 1) are maintaining the basic expression of *eNOS*. As a response to pathological conditions (such as inflammation or TNFα (tumor necrosis factor α)), the expression of *eNOS* is decreased [23]. During recent decades, many regulators of eNOS have been identified: KLF2 and KLF4, MAZ (MYC-associated zinc finger protein), p53, AP-1 (jun proto-oncogene), ELF1 (E74-like ETS transcription factor 1), SP1 (specificity protein 1) and SP3, GATA, and others. Additionally, post-translational modifications regulate the activity of eNOS (phosphorylation on Ser-1177 marks eNOS activation, and Thr-495 inhibition; under high glucose conditions Ser-1177 could be O-glycosylated with a further reduction of eNOS activity) [24].

The decline of the eNOS expression and/or its activity due to inflammation, immune or metabolic damage is the symptom of EC dysfunction and the primary marker of the high atherosclerosis risk. Conversely, maintaining the adequate expression and/or activity of eNOS prevents pathologic vascular remodeling and protects vascular homeostasis in the face of obstructive vascular disease progression [25]. Further, we discuss several recently identified molecular pathways, where *KLF2* is the main activator of the eNOS expression/activity and other genes known to regulate *KLF2* expression.

### 2.1. KLF2-Based eNOS/NO Regulation

The early stages of atherosclerosis progression are associated with the expression of *LOX-1* (lectin-like oxidized low-density lipoprotein receptor-1)—the primary receptor of oxLDL in ECs. OxLDL in the bloodstream bind to LOX-1 on ECs and are internalized into the endothelium transferred to scavenger receptors on macrophages, facilitating foam cell formation and apoptosis of ECs. *LOX-1* expression could be up-regulated in various pathological conditions and stimuli (such as lipopolysaccharide, hypertension, and dyslipidemia) [26]. DF induces *LOX-1* expression via AP-1 (activator protein-1) TF, while KLF2 acts as an inhibitor to AP-1. These results suggest that KLF2 activation is a crucial factor in LOX-1 expression by shear stress, where the knockdown of KLF2 increased the LOX-1 expression in UF, while KLF2 overexpression inhibited the up-regulation of *LOX-1* in DF [27].

Humanin (HN) is a 24-amino acid mitochondrial-derived peptide known for various protective activities: suppress apoptosis, protect against oxidative stress, senescence, and mitochondrial dysfunction, microvascular remodeling, endothelial inflammation, and cell death via various mechanisms [28,29]. Recent data suggest that HN has therapeutic potential for treating high glucose-associated endothelial dysfunction. HN treatment induces the expression of *KLF2* in an ERK5-dependent way and regulates the expression of KLF2 target genes (*eNOS* and *ET-1*). HN also reduces the expression of *VCAM-1* and *E-selectin* and prevents high glucose-induced attachment of the monocyte to ECs [30].

KLF2 bounds to the promoter of *HRD1* (3-hydroxy-3-methylglutaryl reductase degradation) and positively regulated *HRD1* expression. *HRD1* is an E3 ubiquitin ligase involved in ER-associated protein degradation, whose expression was significantly decreased in atherosclerotic intima and by ox-LDL in ECs. Furthermore, overexpression of *HRD1* inhibited the endothelial apoptosis induced by ox-LDL. Mechanically, HRD1 interacts with LOX-1 and promotes its ubiquitination and degradation by the ubiquitin-proteasome system [31].

KLF2-mediated suppression of MiR-155 reduces the pro-inflammatory activation of macrophages. MiR-155, a typical multifunctional microRNA, plays important roles in immunity and inflammation, particularly in the inflammatory responses of macrophages, suggesting a potential role for MiR-155 also in atherogenesis [32]. MiR-155 is enriched in ox-LDL-induced EC-extracellular vesicles and leads to enhanced monocyte activation by shifting the monocyte/macrophage balance from anti-inflammatory M2 macrophages towards pro-inflammatory M1 macrophages. However, endothelium-derived extracellular vesicles from KLF2-expressing HUVECs have reduced levels of MiR-155, decreased pro-inflammatory responses, and enhanced immunomodulatory responses, resulting in suppressed monocyte activation, reduced atherosclerotic lesion formation, and decreased levels of pro-inflammatory M1 macrophages and increased levels of anti-inflammatory M2 macrophages. Thus, KLF2 inhibits inflammation-associated MiR-155 in ECs, which is crucial for the selective growth and differentiation of macrophages towards an anti-inflammatory M2-like phenotype [33].

KLF2 regulates the differentiation of endothelial progenitor cells to ECs induced by shear stress. Additionally, KLF2 binds to the promoter and increases expression of the von Willebrand factor (*VWF* gene), which encodes a glycoprotein involved in hemostasis, adhesion of platelets to sites of vascular injury, and the transport of various proteins in the blood [34].

NRFf2 (nuclear factor, erythroid 2 like 2) and HO-1 (heme oxygenase 1) are key factors regulating oxidative stress in the body. NRF2 is a transcription factor that plays a vital role in activating various defense mechanisms against OS and exogenous toxic substances. Under hypoxia and reoxygenation conditions, the expression levels of *NRF2* and *HO-1* were increased compared to control cells. The overexpression of KLF2 increased the cell viability, eNOS activity, and NO levels in an NRF2/HO-1-dependent way, the rations of crucial eNOS cofactor tetrahydrobiopterin (BH4/BH2) and antioxidant glutathione/oxidized glutathione, with subsequent down-regulation of the generated O2^•−^ and ONOO^−^ [35].

Recently, it was shown that EPO (erythropoietin), a glycoprotein secreted by the fetal liver and adult kidney, a crucial hematopoietic factor known to promote the physiological functions of red blood cell proliferation, differentiation, and maturation, also activates AMPK (protein kinase, AMP-activated, alpha 2 catalytic subunit)-KLF2 signalling pathway and regulates *HIF-1α* (hypoxia inducible factor 1 subunit alpha) and *eNOS* expression to promote new vascular development after cerebral ischemia [36].

**Figure 1:**
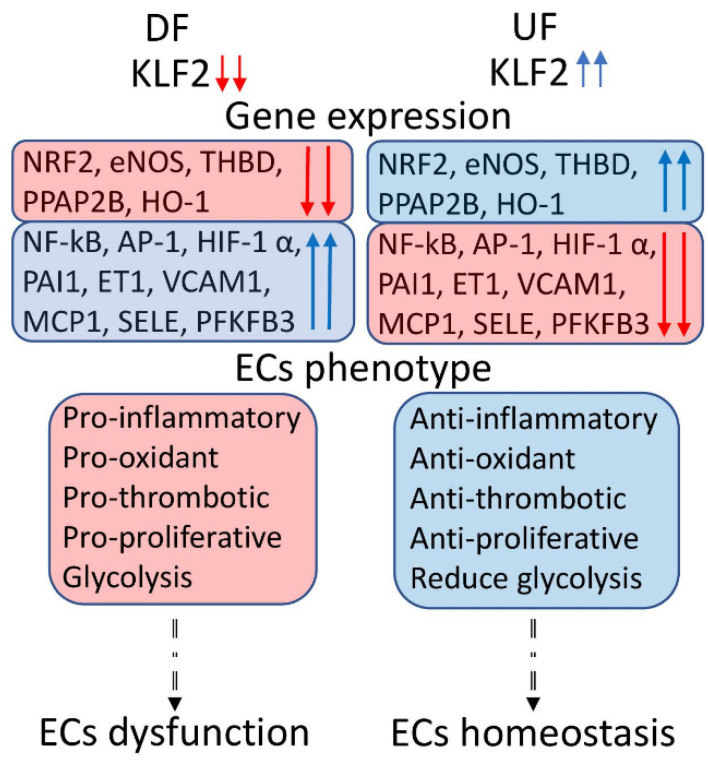
The role of KLF2 (Kruppel like factor 2) in ECs (endothelial cells) dysfunction and homeostasis. KLF2 is the major MSTF regulated by shear stress: down-regulated by disturbed flow (DF) and up-regulated by unidirectional laminar flow (UF). Gene expression boxes represent major genes regulated by KLF2. Red arrows show the down-regulation, and up-regulation is indicated by blue arrows. Further, gene expression resulted in ECs phenotype. DF exposed ECs with down-regulated KLF2 show a pro-inflammatory, pro-oxidant, pro-proliferative response and enhanced glycolysis, resulting in ECs dysfunction. On the contrary, UF exposed ECs with up-regulated KLF2 show anti-inflammatory, antioxidant, and antiproliferative phenotype and reduced glycolysis, leading to ECs stabilization and vascular homeostasis.

Several important interactions (KLF2 with NRF2, NF-kB (nuclear factor kappa B subunit 1) with HIF-1α) have created a regulatory circuit with many unknown relations. For example, KLF2 potentially could inhibit the expression and activity of *HIF-1α*, while HIF-1α also participates in DF-mediated KLF2 down-regulation [37]. Further, the potential interaction between KLF2 and NRF2 is crucial in light of redox-independent regulation of inflammatory and metabolic gene expression by NRF2 [38]. Thus, such complex relations and interconnections between different regulators and metabolic pathways require further investigation and could provide a new direction in our understanding of the effects of hemodynamic forces on endothelial function (Figure 1).

### 2.2. Genes Regulating KLF2

Omentin-1 is an important adipokine mainly secreted by stromal vascular cells in visceral adipose tissue has been reported to be involved in different types of physiological processes, including anti-inflammatory response, cardiovascular action, and redox homeostasis. In addition, Omentin-1 activates the Akt signaling pathway, thus, regulating downstream glucose metabolism and could be beneficial for enhancing insulin-stimulated glucose uptake; decreased serum omentin-1 is also associated with inflammatory bowel disease [39]. Importantly, omentin-1 has been shown to act against atherosclerosis and ischemia-induced revascularization, improve heart damage and functions after reperfusion therapy in patients with acute myocardial infarction, ameliorate acute ischemic injury and stroke, attenuated neointimal formation after arterial injury, and suppressed vascular smooth muscle cells [40,41].

Recently it was found that omentin-1 significantly reduced the attachment of the leukocyte cells to HUVECs (human umbilical vein endothelial cells) and prevented the expression of cell adhesion molecules (*VCAM-1* and *E-selectin*). Importantly, omentin-1 in a p53-dependent way restored ox-LDL-mediated reduction of KLF2 and promoted the expression of KLF2 downstream targets—*eNOS* and *PAI-1*. ox-LDL (oxidation of low-density lipoproteins) play an essential role in endothelial dysfunction and the pathological progression of atherosclerosis, which is caused by a leukocyte attachment to endothelial surfaces. Thus, suggesting the high potential of omentin-1 as a therapeutic target in preventing endothelial dysfunction and atherosclerosis [42].

*GPR81* (G coupled-protein receptor 81, or HCA1 (hydroxycarboxylic acid receptor 1)) is a recently identified lactate-activated athero-protective gene. DF down-regulates the expression of *GPR81* in ECs, while its activation in DF stressed ECs leads to reduced OS, down-regulates the expression of inflammatory cytokines (IL-6, IL-8, and MCP-1) and HMGB1 (high mobility group box 1), which is involved in DNA organization, suppress VCAM-1 and E-selectin secretion, and promote ERK5-mediated *KLF2* expression [43].

*IRF2BP2* (interferon regulatory factor 2-binding protein 2) was found to attenuate macrophage-mediated inflammation and susceptibility to atherosclerosis. Mechanically, IRF2BP2 binds to KLF2 and up-regulates *KLF2* expression, resulting in HUVECs protection against ox-LDL-induced inflammation, endothelial-to-mesenchymal transition, and endothelial dysfunction [44].

*A20* (or tumor necrosis factor alpha-induced protein 3) is a zinc finger protein and ubiquitin-editing enzyme known to inhibit NF-kB activation as well as TNFα-mediated apoptosis and involved in the cytokine-mediated immune and inflammatory responses. A20 overexpression in ECs increases eNOS transcription in an ERK5/KLF2-dependent way and promotes eNOS activating phosphorylation. This effect sustains TNFα-mediated eNOS downregulation, thus preventing ECs dysfunction during inflammation [45].

Patients with chronic kidney disease show early signs of endothelial dysfunction, which manifests as decreased NO levels, increased oxidative stress, inflammatory activation, and eventual apoptosis or necrosis. As shown, KLF2 expression is suppressed by an AGE (advanced glycation end product) and induces endothelial dysfunction. In particular, the migration of the p65 subunit of NF-κB to the nucleus is necessary to suppress *KLF2* transcription [46].

*FOXP1* (forkhead box P1) is a TF containing both DNA-binding- and protein–protein binding-domains and involved in KLF2-mediated athero-protection of ECs via the regulation of endothelial inflammasome. KLF2 regulates Foxp1 and directly regulates endothelial inflammasome components (such as Nlrp3 (NLR family pyrin domain containing 3), caspase-1 and IL-1β. In human patients with atherosclerosis and athero-susceptible endothelium of mouse models, the expression of *FOXP1* was down-regulated. However, the ECs-specific Foxp1 overexpression in Foxp1^ECTg^; Apoe^KO^ hyperlipidaemic mouse model resulted in reduced atherosclerotic lesion formation with less monocyte infiltration. Thus, suggesting the role of shear stress in down-regulation of ECs’ Foxp1 (via KLF2 repression) and inflammasome activation, promoting atherosclerosis [47].

*BDNF* (brain-derived neurotrophic factor) plays an athero-protective role in vascular ECs, acting via KLF2/HK1 (hexokinase 1)-mediated modulation of glucose metabolism. HK1 is localized to the outer membrane of mitochondria, phosphorylate glucose to produce glucose-6-phosphate and is involved in innate immunity and inflammasome activation [48]. Patients with coronary artery disease had lower BDNF and increased lactate levels than healthy people. Thus, the application of recombinant BDNF resulted in reduced ox-LDL-induced NLRP3 inflammasome formation and ECs pyroptosis; also, levels of caspase-1, IL-1β, IL-18, and released lactate dehydrogenase were reduced. KLF2 interacts with HK1 and HK1 overexpression cause NLRP3 inflammasome formation. Additionally, the BDNF/KLF2 pathway preserves the mitochondrial membrane potential, ATP production, electron transport chain processing, intracellular ROS generation, and oxygen consumption rate [49].

In total, emerging evidence indicates that endothelial KLF2 is the major MSTF that regulates vascular homeostasis EC metabolism and represents a promising therapeutic target for atherosclerosis treatment and prevention by pharmacological intervention (Figure 1). Recently, other members of the KLF family have been identified, and their involvement in different diseases described [50,51]. While other members of the KLF family also exhibit anti-inflammatory functions, it would be interesting to assess their mechanosensitive properties and potential interaction with KLF2 in the regulation of EC metabolism and athero-protective functions. Furthermore, antagonistic relations between NF-kB and KLF2 require further attention. For example, in a cooperative effect with histone deacetylases (HDAC4 and HDAC5), NF-kB inhibits MEF2 (myocyte enhancer factor 2C) and its target KLF2. Simultaneously, MEF2 could stimulate *KLF2* expression and inhibit cytokine-induced NF-kB activation [52]. Thus, suggesting that many shear stress-activated TFs interact with each other in ECs, and these crosstalks between TFs could stimulate different responses to different patterns.

## 3. Repurposing Drugs to Treat Atherosclerosis (Acting on KLF2)

Many long-time known and well-studied drugs were recently shown to provide diverse beneficial effects on other non-target systems. This approach, defined as “drugs repurposing”, allows a more effective and quick way to treat different diseases without developing new drugs. For example, cardioprotective properties were assigned for anti-diabetic medications when used in patients with high CVDs risk, even in non-diabetic patients [53]. It is known that diabetes is one of the significant risk factors for developing CVDs, chronic kidney disease, blindness, and stroke. Impaired blood glucose levels are crucial for the development and progression of heart failure and CVDs in diabetes patients. Application of glucose-lowering drugs such as DPP4i (dipeptidyl-peptidase 4 inhibitors), SGLT2i (sodium-glucose transporter 2 inhibitors) and GLP1-RA (glucagon-like peptide-1 receptor agonists) was beneficial for cardioprotection of both diabetic and non-diabetic patients. However, their cardioprotective mechanisms are mostly unknown and require further investigation [54]. Further, we describe several well-known drugs with recently identified athero-protective effects based on the KLF2 activation.

Montelukast is a selective, reversible CysLT1 (cysteinyl leukotriene receptor 1) receptor antagonist, known for its antioxidant, anti-inflammatory and anti-apoptotic effects and used to treat various inflammatory diseases [55]. In the early stages of atherosclerosis, Montelukast was shown to promote *KLF2* expression in an ERK5-mediated way, which resulted in a reduction of oxLDL-induced monocyte adhesion to HUVECs and suppression of adhesion molecule genes expression (*VCAM-1* and *E-selectin*), thus, confirming athero-protective properties [56].

Substance P, a member of the tachykinin peptides family, binds to the NK-1R (neurokinin-1 receptor) and plays an essential role in inflammation and pain, regulating vessel elasticity, blood pressure, and heart frequency [57]. It was shown that *NK-1R* is expressed in ECs and induced by oxLDL, thus being involved in the pathological progression of cardiovascular disease. Presence of aprepitant, a substance P-like selective NK-1R antagonist, acting via ERK5/KLF2 axis, protects ECs from ox-LDL-induced inflammatory response and injury, restores normal eNOS/NO levels [58].

Azilsartan is an angiotensin II type 1 receptor blocker, developed for the treatment of hypertension, with wide antioxidant and anti-inflammatory properties (in particular, increase ECs eNOS activation, regulate inflammatory cytokines expression, inhibit lipid peroxidation, and ROS generation) and preserve mitochondrial function (inhibit mitochondrial swelling, maintain ATP production, reduce cytochrome c release, and increase mitochondrial membrane potential) [59,60]. Recently it was found that azilsartan treatment in KLF2-dependent way alleviates ox-LDL-induced effects: suppresses the expression of *LOX-1*, *CXCL-1*, and *MCP-1*, reverse *eNOS* expression and NO production, normalizes endothelial monolayer permeability and occludin expression. Thus, the suggestion is that azilsartan could be used to treat endothelial dysfunction and cardiovascular diseases, including atherosclerosis [61].

Laquinimod, an experimental immunomodulator with high clinical potential [62], also has a potential application as an anti-atherosclerotic agent. Laquinimod increases *KLF2* expression in an ERK5-dependent way reduces the expression of adhesion molecules (VCAM-1 and E-selectin) and central inflammatory cytokines and chemokines (IL-6, MCP-1, and HMGB1) [63].

A widely used pharmacology drug solubilizer and penetration enhancer, NMP (N -methyl-2-pyrrolidone), also has anti-inflammatory properties, confirmed in ApoE^−/−^ mouse model of atherosclerosis. The molecular mechanism of NMP’s activities relies on *KLF2* up-regulation and down-regulation of adhesion molecules, pro-inflammatory cytokines, and chemokine receptors [64].

Vorinostat (suberanilohydroxamic acid or SAHA) is a histone deacetylase inhibitor used as an approved treatment of cutaneous T cell lymphoma and acute myeloid leukaemia, and potentially could be used against several other diseases [65]. SAHA shows KLF2-dependent anti-atherosclerotic effects through reduced monocyte adhesion to ECs, inhibited endothelial inflammation in vitro, and high-fat diet-induced atherosclerotic lesion development in ApoE^−/−^ mice model [66].

### 3.1. Anti-Diabetic Drugs

Liraglutide is a licensed type 2 diabetes drug, GLP-1 (glucagon-like peptide 1) analogue, due to its antioxidative and anti-inflammatory properties against TNF-α-induced injury and NF-kB signaling in ECs, and also possesses cardiovascular protection properties [67]. Liraglutide induces ERK5 phosphorylation and ameliorates ox-LDL-induced reduction of the *KLF2* expression. Subsequent effects of liraglutide are KLF2-mediated; it normalizes *eNOS* expression and NO release, inhibits *E-selectin*, and *VCAM-1* expression, prevents ox-LDL-induced attachment of monocytes adhesion to ECs and ameliorates endothelial monolayer permeability and *Occludin* expression [68].

GPR120 (free fatty acid receptor 4) is a receptor for free fatty acids (including omega-3), also known to suppress anti-inflammatory responses and insulin-sensitizing [69,70]. Thereby, GPR120 agonists (GW9508 and TUG-891) have been shown to mitigate ox-LDL-induced ECs dysfunction by suppressing OS and inflammation, inhibiting ROS production and expression of pro-inflammatory cytokines, VCAM-1 and E-selectin, induce KLF2 expression [71].

#### The Role KLF2 in Autophagy Activation

Similarly, another crucial diabetes drug metformin was shown to provide an anti-atherogenic effect up-regulating KLF2-mediated autophagy. Experiments on high-fat diet ApoE^−/−^ mice suggested that metformin enhances autophagy and subsequently inhibits foam cell formation and cellular apoptosis, reducing plaque stability and general athero-protective effect [72].

Autophagy is the primary cellular system intended to recycle and process malfunction organelles and proteins to maintain cellular homeostasis. During atherosclerosis and other CVDs, the primary function of autophagy is to maintain cell function and prevent increased pressure from cell death and senescence [73]. Recently, the role of KLF2 in the activation of autophagy in smooth muscle cells during AAA (abdominal aortic aneurysms) was shown [74]. Abdominal aortic aneurysms are among the leading healthcare concerns worldwide because they are asymptomatic, challenging to diagnose and result in a high mortality rate, especially among the aged population. On the molecular level, AAA was characterized by an early elevation of pro-inflammatory cytokines (IL-1β, IL-6, IL-17, IL-23, MCP-1, TNFa, and IFNγ (interferon gamma)), gradual elastin and collagen degradation with further smooth muscle cell apoptosis, necrosis, and autophagy, resulted in the chronic, thrombotic process common for most human AAAs [75]. It was shown that KLF2 could bind *BECN1* promoter (also known as ATG6), one of the phosphatidylinositol-3-kinase autophagy regulators; thus, KLF2 plays a role in aneurysm formation via direct activation of autophagy genes [74]. The role of KLF2 in autophagy regulation via BECN1 is not specific only for smooth muscle cells because the same mechanism was studied during osteoclastogenesis [76] and osteoblast differentiation of dental pulp derived stem cells [77], suggesting a universal type of interaction.

## 4. Plant Compounds

Atherosclerosis is one of the major CVDs which occur through multiple molecular mechanisms and is associated with different metabolic disorders. Conventional pharmacological therapies used to treat atherosclerosis are often associated with mild to severe side effects. Thus, natural compounds, primarily plant-based bioactive compounds, have been suggested as a potential solution to prevent and treat atherosclerosis and associated diseases [78]. Further, we discuss several natural compounds (such as leaves extracts, flavonoids, and polyphenols) with defined mechanisms of action on KLF2 and used for atherosclerosis treatment.

Natural polyphenol resveratrol, known for its diverse beneficial effects and wide application in laboratories and clinically, also has athero-protective properties. Resveratrol up-regulates *KLF2*, down-regulates inflammatory factors (*ICAM-1* (intercellular adhesion molecule-1) and *MCP-1*) and normalize NO secretion [79].

Cardio- and athero-protective effects were also shown for an ethanol extract of soy leaf (ESL) on *LDLR*^−/−^ high-cholesterol diet mice model. ESL supplementation leads to decreased expression of aortic inflammation and ECs dysfunction markers such as fractalkine (C-X3-C motif chemokine ligand 1), MMP-9 (matrix metallopeptidase 9), MCP-1, IL-1β, IL-6, TNF-α, ICAM-1, and VCAM-1. On the other side, the expression of *KLF2* and eNOS was increased. In total, ESL supplementation resulted in reduced aortic lesion formation, suggesting high atheroprotective properties [80].

Tannic acid, a polyphenolic compound, can induce ERK5-dependent expression of *KLF2* in ECs. Functionally, tannic acid treatment of the lung ECs isolated from Klf2^+/+^ and Klf2^+/−^ mice resulted in reduced expression of the adhesion molecule *VCAM1* [81].

Puerarin is a bioactive herbal isoflavone known for its antioxidation and anti-inflammation properties, used to protect from atherosclerosis and cardiovascular diseases [82]. The defined molecular mechanism of Puerarin activities relies on ERK5-dependent up-regulation of *KLF2* expression and its target genes (*eNOS* and *THBD*) and reduce the expression of adhesion-related genes (*IL-8*, *MCP-1*, *VCAM-1*, and *ICAM-1*). In vivo experiments on ApoE^−/−^ mice on a high-fat diet treated with puerarin had significantly reduced atherosclerosis lesions in the aorta [83].

DHL (dehydrocostus lactone) is a natural compound derived from the *Saussurea lappa* costus plant. DHL treatment up-regulates *KLF2* expression and down-regulates expression of *VCAM-1* and *E-selectin*, ECs release of pro-inflammatory cytokines and chemokines (HMGB1, TNF-α, and MCP-1). Thus, DHL could serve as a prophylactic or therapeutic treatment against atherosclerosis [84].

Similarly, Ginkgo biloba extract exhibits KLF2-mediated antiplatelet and anticoagulant activities via up-regulation of thrombomodulin and tissue-type plasminogen activator genes expression in ECs [85].

Artesunate, a derivative of plant-extracted malaria drug artemisinin, was shown to reduce the formation of atherosclerotic plaques, acting through increased expression of *KLF2* and *LPL* (lipoprotein lipase)—the central triglyceride hydrolyzing enzyme, which deficiency leads to atherosclerosis [86]. In vitro experiments on VSMC (vascular smooth muscle cells) suggest that ART increased *LPL* expression and inhibition of NRF2 blocked the binding of TCF7L2 (transcription Factor 7 Like 2) to the *LPL* promoter region. For in vivo experiments, HFD fed ApoE^−/−^ mice were injected with artesunate, which resulted in decreased atherosclerotic plaque formation, increased VSMC number, and *LPL* expression within atherosclerotic plaques. Thus, the ART-activated molecular mechanism in VSMCs involves up-regulation of the KLF2/NRF2/TCF7L2 pathway, leading to increased *LPL* expression and anti-atherosclerotic effect [87].

Phloretin, a major phenolic compound found in apples and strawberries, could ameliorate diabetic atherosclerosis via the KLF2-eNOS axis in ApoE^−/−^ mice. Additionally, phloretin benefits lipid metabolism, normalizing triglycerides and LDL-cholesterol levels in a KLF2-dependent way [88].

Betulinic acid (BA) is a natural compound of pentacyclic triterpene isolated from tree Betula and exhibiting broad biological activities against inflammation, cancer, diabetes, cardiovascular diseases, and atherosclerosis [89]. BA up-regulates *eNOS* expression and NO synthesis in HDAC5/ERK5/KLF2-dependent way. BA also increases levels of intracellular Ca^2+^ via TRPC1 (capacitative calcium channel protein Trp1), a non-selective channel permeable to calcium and other cations, which further resulted in activation of AMPK, CaMKIIα (calcium/calmodulin dependent protein kinase II alpha) and CaMKKβ (calcium/calmodulin dependent protein kinase kinase 2). In total, due to described biological activities of BA has a high potential in the treatment of endothelial dysfunction and cardiovascular diseases such as hypertension, ischemic stroke, and atherosclerosis [90].

**Figure 2:**
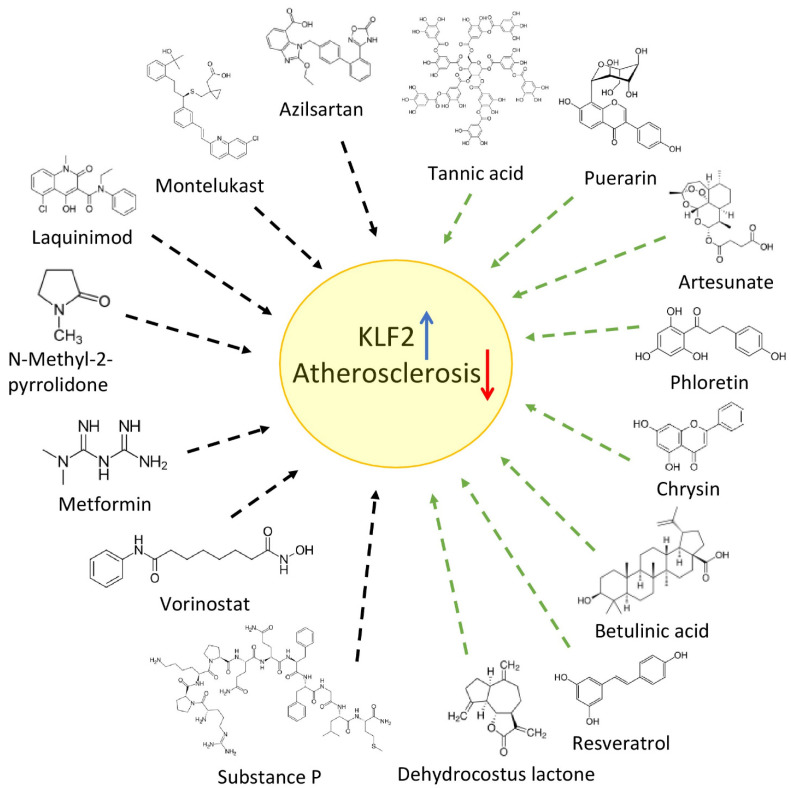
Summary of recently identified drugs and natural plant-based bioactive compounds that could be used for atherosclerosis treatment. Known drugs (black arrows) and plant compounds (green arrows) can up-regulate KLF2 expression (blue arrow) and, with defined athero-protective properties (red arrow), act via different mechanisms and molecular pathways (further details are provided in the text).

Natural flavonoid chrysin, which is presented in several plants and fruits, has anti-inflammatory and cardioprotective properties [91]. Recently it was defined that the atheroprotective properties of chrysin are mediated via suppression of the MiR-92a [92], which is the primary regulator of KLF2 and KLF4 [93] (Figure 2).

Atherosclerosis development and progression are usually associated with other morbidities, complications, and metabolic disorders (such as diabetes, insulin resistance, obesity, non-alcoholic fatty liver disease, and others). However, with a solid understanding of mechanistic and molecular details involved in the development of atherosclerotic lesions, it is promising to search for a more practical application of known medications and natural bioactive compounds to modify the course of atherosclerosis to a more favorable one. In the case of repurposed drugs, the main advantages are known molecular mechanism of action, tolerance levels, pharmacokinetics, and side effects, which should be verified on the atherosclerosis model systems. Plant-based bioactive compounds are usually well-tolerated and provide wide antioxidant, antifibrotic, anti-inflammatory, antitumor, and cardioprotective effects. However, to fully assess the effectiveness of the repurposed drugs and plant-based bioactive compounds for treating atherosclerosis and associated disorders, further research is required to explore their therapeutic activities at cellular levels.

## 5. Conclusions

Atherosclerosis is the devastating underlying cause of CVDs, and it preferentially develops at arterial regions exposed to DF, while the areas of UF are protected and less prone for it. KLF2 is one of the best-studied MSTFs activated by mechanical forces and involved in the regulation of essential aspects of endothelial function, such as oxidative stress, mitochondrial function, glucose metabolism, cell proliferation, senescence, vascular inflammation, and vascular tone, thus, playing a critical role in atherosclerosis and CVDs development. Emerging studies show that KLF2 represents a promising therapeutic target for pharmacological intervention to prevent and treat atherosclerosis. In this review, we present a comprehensive overview of the role of KLF2 in atherosclerosis development and progression. We also highlight future directions for applying repurposed drugs and natural plant-based bioactive compounds in developing novel therapeutic strategies targeting KLF2 in atherosclerosis treatment and prevention.

## Data Availability

Not applicable.

## Abbreviations

CVDs	cardiovascular diseases
VSMCs	vascular smooth muscle cells
LDL	low-density lipoproteins
DF	disturbed flow
UF	unidirectional laminar flow
MSTFs	mechanosensitive transcription factors
THBD	thrombomodulin
PAI1	plasminogen activator inhibitor-1
SELE	E-selectin
ET1	endothelin 1
MCP-1	monocyte chemoattractant protein
VCAM1	vascular cell adhesion molecule
PPAP2B	phosphatidic acid phosphatase type 2B
PFKFB3	6-phosphofructo-2-kinase/fructose-2,6-biphosphatase-3
NO	nitric oxide
ECs	endothelial cells
eNOS	endothelial nitric oxide synthase
TGFB1	transforming growth factor beta 1
TNFα	tumor necrosis factor α
MAZ	MYC-associated zinc finger protein
AP-1	jun proto-oncogene
ELF1	E74-like ETS transcription factor 1
SP1	specificity protein 1
LOX-1	lectin-like oxidized low-density lipoprotein receptor-1
AP-1	activator protein-1
HN	humanin
HRD1	3-hydroxy-3-methylglutaryl reductase degradation
NRF2	nuclear factor, erythroid 2 like 2
HO-1	heme oxygenase 1
AMPK	protein kinase, AMP-activated, alpha 2 catalytic subunit
HIF-1α	hypoxia-inducible factor 1 subunit alpha
NF-kB	nuclear factor kappa B subunit 1
ox-LDL	oxidation of low-density lipoproteins
GPR81	G coupled-protein receptor 81
HCA1	hydroxycarboxylic acid receptor 1
HMGB1	high mobility group box 1
IRF2BP2	interferon regulatory factor 2-binding protein 2
AGE	advanced glycation end product
FOXP1	forkhead box P1
Nlrp3	NLR family pyrin domain containing 3
BDNF	brain-derived neurotrophic factor
HK1	hexokinase 1
HDAC4	histone deacetylases 4
MEF2	myocyte enhancer factor 2C
CysLT1	cysteinyl leukotriene receptor 1
NMP	N-methyl-2-pyrrolidone
SAHA	suberanilohydroxamic acid
GLP-1	glucagon-like peptide 1
GPR120	free fatty acid receptor 4
AAA	abdominal aortic aneurysms
ICAM-1	intercellular adhesion molecule-1
ESL	ethanol extract of soy leaf
MMP-9	matrix metallopeptidase 9
LPL	lipoprotein lipase
VSMC	vascular smooth muscle cells
BA	betulinic acid
TRPC1	capacitative calcium channel protein Trp1
CaMKIIα	calcium/calmodulin-dependent protein kinase II Alpha
CaMKKβ	calcium/calmodulin-dependent protein kinase kinase 2
